# Compromised External Validity: Federally Produced *Cannabis* Does Not Reflect Legal Markets

**DOI:** 10.1038/srep46528

**Published:** 2017-04-19

**Authors:** Daniela Vergara, L. Cinnamon Bidwell, Reggie Gaudino, Anthony Torres, Gary Du, Travis C. Ruthenburg, Kymron deCesare, Donald P. Land, Kent E. Hutchison, Nolan C. Kane

**Affiliations:** 1 University of Colorado Boulder, Department of Ecology and Evolutionary Biology, Boulder, CO 80309-0334, USA; 2 University of Colorado Boulder, Institute of Cognitive Science, Boulder, CO 80309-0334, USA; 3 Steep Hill Labs Inc. 1005 Parker Street, Berkeley, CA 94710, USA; 4 University of Colorado Boulder, Department of Psychology and Neuroscience, Boulder, CO 80309-0334, USA

## Abstract

As the most widely used illicit drug worldwide, and as a source of numerous under-studied pharmacologically-active compounds, a precise understanding of variability in psychological and physiological effects of *Cannabis* varieties is essential. The National Institute on Drug Abuse (NIDA) is designated as the sole legal producer of *Cannabis* for use in US research studies. We sought to compare the chemical profiles of *Cannabis* varieties that are available to consumers in states that have state-legalized use versus what is available to researchers interested in studying the plant and its effects. Our results demonstrate that the federally-produced *Cannabis* has significantly less variety and lower concentrations of cannabinoids than are observed in state-legal U.S. dispensaries. Most dramatically, NIDA’s varieties contain only 27% of the THC levels and as much as 11–23 times the Cannabinol (CBN) content compared to what is available in the state-legal markets. Research restricted to using the current range of federally-produced *Cannabis* thus may yield limited insights into the chemical, biological and pharmacological properties, and medical potential of material that is available in the state markets. Investigation is urgently needed on the full diversity of *Cannabis* chemotypes known to be available to the public.

The United States has witnessed enormous changes concerning public acceptance of Cannabis, or marijuana in the vernacular. From 2002 to 2012, the number of individuals reporting past year use more than doubled, across all genders, ethnicities and socioeconomic status^1^, in tandem with the rise in individual-states’ relaxation or ending of Cannabis prohibition. Considering these changes on the cultural, political, and legal fronts, research on the effects of Cannabis products available through legal outlets in the United States is urgently needed.

The Cannabis plant is unique in producing a diversity of cannabinoids and terpenoid chemical compounds that interact with the endocannabinoid system in the brain and nervous systems^2^. One of the primary cannabinoids produced, Δ-9-tetrahydrocannabinolic acid (THCA), is converted to the neutral form Δ-9-tetrahydrocannabinol (THC) when heated, as in smoking or cooking. THC interacts with the endocannabinoid system producing a wide range of physiological and neurological effects. Studies have found that Cannabis’ effects on mood, reward, and cognitive dysfunction appear to follow a dose dependent function based on the THC content^3^. Due to this and other purported psychotropic effects, THCA has been actively selected for by both the licit^4^ and illicit^5^ Cannabis markets, and varieties containing more than 30% THCA by weight have been cultivated^6^.

In addition to THC, Cannabis’ effects are likely related to a number of other compounds^7^,^8^, including nearly 74 different cannabinoids^9^ present at varying ratios across varieties. For example, cannabidiolic acid (CBDA), is converted to cannabidiol (CBD) when heated, which in turn appears to attenuate the dose effects of THC such as anxiety and psychosis^10^,^11^,^12^,^13^,^14^,^15^ and may have other benefits^11^,^12^,^14^,^15^,^16^,^17^,^18^,^19^,^20^,^21^. Demand for high CBDA plants has been increasing^5^, due to potential therapeutic uses for cancer^22^ and epilepsy^23^,^24^. Other important cannabinoids produced by the plant include cannabigerol (CBG)^25^, cannabichrome (CBC)^26^, and Δ-9-tetrahydocannabivarin (THCV)^27^.

Because research universities across the United States rely heavily on national grants and must verify compliance with federal law, investigators at these institutions are restricted to using the only federally-legal source of Cannabis plant material: the National Institutes of Health/National Institute on Drug Abuse (NIDA). Thus, the results and conclusions of nearly all published US laboratory studies as to Cannabis effects in humans rely exclusively on the material represented in the NIDA sample set, often administered in a laboratory setting^28^,^29^,^30^. At the same time, dispensary-grade Cannabis available to consumers in individual-state-regulated markets in the U.S. is becoming increasingly potent and diverse. Varieties differ substantially in potency and cannabinoid content, and hence, are likely to differ in terms of their potential effects in specific patients/indulgers^4^. Varieties bred for high THCA content are thought to result in greater levels of intoxication as well as differing psychological and physiological effects compared to varieties bred for high CBDA content. In the US, dispensaries must apply for either a medical or recreational license or both to supply Cannabis for either or both purposes. Even though the preference of recreational or medical consumers is not known, research has found that the Cannabis from the black market has increased in THC content^4^,^5^. Although no information about the preference of cannabinoid content for medical users exists, high CBDA varieties are bred for medical purposes^5^.

Accordingly, NIDA has recently developed plant material with varying cannabinoid profiles (chemotypes) for research purposes, but the extent to which this federally-produced and exclusively-sanctioned-for-research Cannabis is consistent with the range of Cannabis chemotypes produced in the private market is not clear.

To address the critical question of whether the potency and variety of NIDA-provided Cannabis reflects products available to consumers through state-regulated markets, we compared the cannabinoid profile variation among plants from four different US cities (Denver, Oakland, Sacramento, and Seattle; cannabinoid data provided by Steep Hill Labs Inc.) representing three of the states where Cannabis is legal for medical or recreational reasons, to the range of cannabinoid contents of plants supplied for research purposes by NIDA, using the data publicly available on their website^31^.

## Results

**Figure 1:**
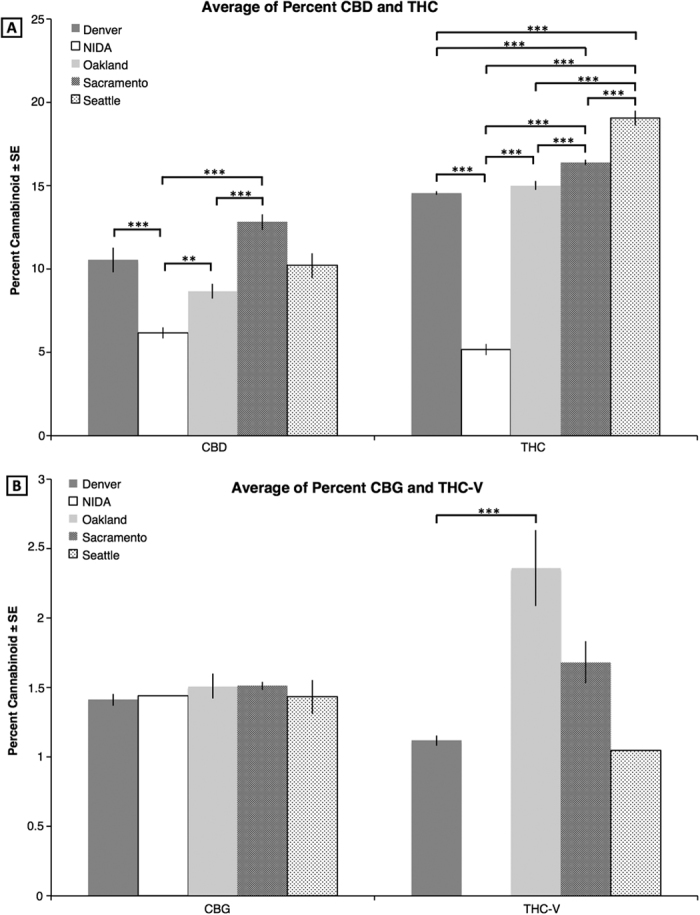
Average percent cannabinoids for five different locations. (**A**) CBD (N = 313) and THC (N = 2923). (**B**) CBG (N = 411) and THC-V (40). Significant values between the comparisons are given in the horizontal bars above: ***P < 0.001; **P < 0.01; and *P < 0.05.

NIDA differs from all other locations except Seattle in production of CBD (Fig. 1A), and differs significantly from all other locations in production of THC. NIDA has the lowest CBD and THC percent with a mean and s.d. of 6.16 ± 2.43%, and 5.15 ± 2.60% respectively. Sacramento has the highest percent CBD with 12.83 ± 4.73% and Seattle has the highest percent THC with 19.04 ± 4.43%. There are significant differences between the percent of both CBD and THC between US city locations, in addition to differences with NIDA (Fig. 1A).

CBG production does not differ in any location. *Cannabis* plants from all five sources produce very little CBG, particularly NIDA with only a single sample having more than 1% CBG (Fig. 1B). THC-V is also produced in low quantities in all locations. The only statistically significant difference is between Denver, whose mean and s.d is 1.12 ± 0.13%, and Oakland 2.35 ± 0.68% (P < 0.001; Fig. 1B). Importantly, Seattle has only one (1) sample and NIDA lacks any plants that produce more than 1% THC-V.

**Figure 2:**
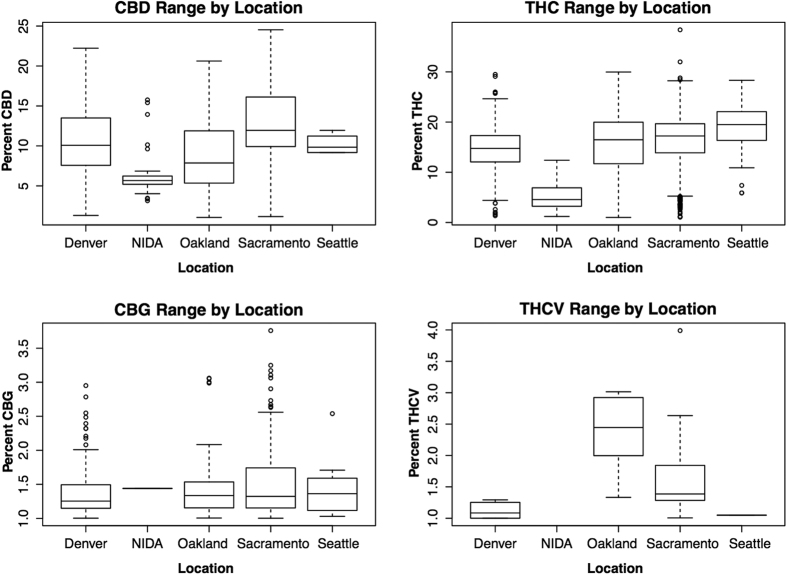
Median and range for cannabinoids by location. Median (line within the box), 25^th^ and 75^th^ percentile (bottom and top of the box respectively), and range (bars outside the box). Outliers are dots outside the box and range. The Y axis differs by panel.

**Figure 3:**
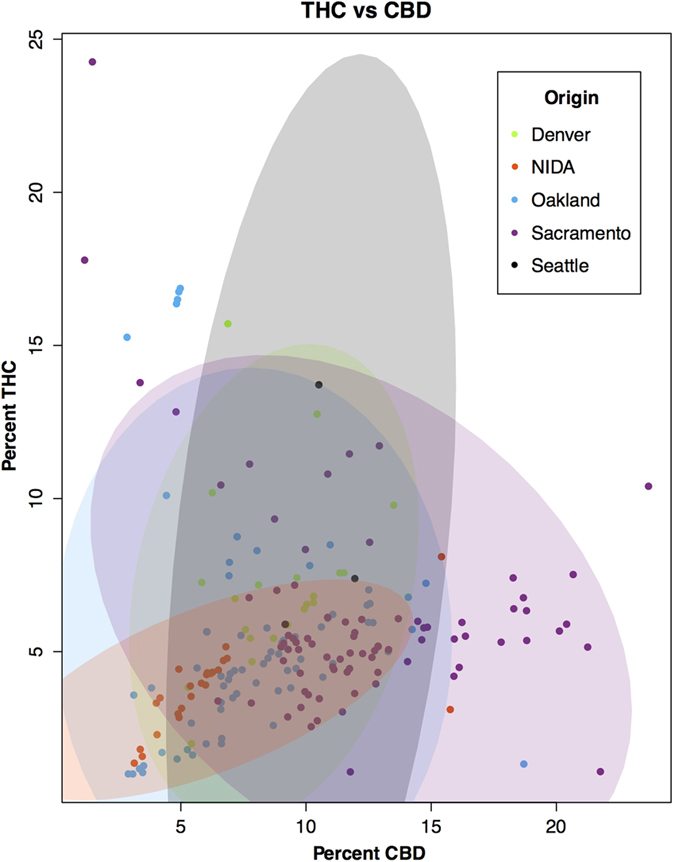
The diversity and variability of *Cannabis* samples across sites in terms of THC, CBD. The ellipses represent 95% confidence (N = 1152).

Two analyses were used to examine the phytochemical diversity found in each location. In the first analysis, the variability and range of each cannabinoid was calculated (Fig. 2). This analysis shows that for three of the four cannabinoids, NIDA has the lowest variability. In addition, the potencies of THC and CBD across sites (Fig. 3) suggest a greater diversity in both potency and ratio in the private market. In other words, the federal varieties show limited diversity in the total cannabinoid levels, in the cannabinoids that are present, and in the ratio of cannabinoids (Figure S1).

**Figure 4:**
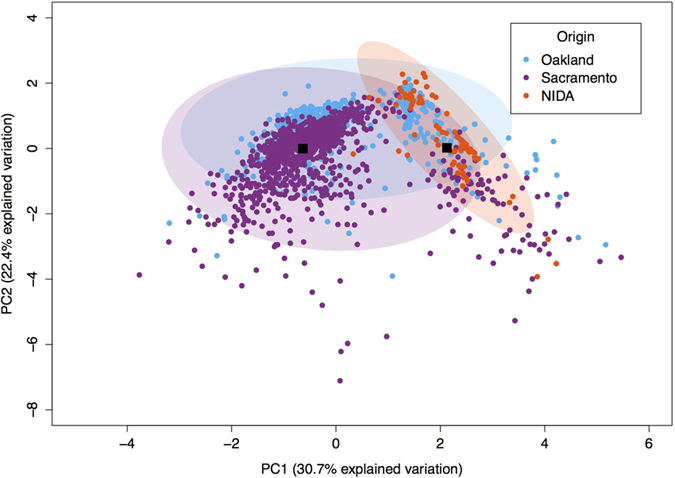
PC1 vs PC2 for three locations. Most of the points from the two main PC axes overlap demonstrating similarities between the three locations in their content. The black boxes represent the means of the two clusters after the k-means analysis.

The Principal Component Analysis (PCA, Fig. 4) shows that 53.1% of the overall cannabinoid variation is explained by PC1 and PC2, with PC1 explaining 30.8% and PC2 explaining 22.3%. PCA shows that the overall cannabinoid content in Oakland and Sacramento is very similar, since the points overlap with each other, even though Sacramento has more variation. Most of NIDA’s samples cluster within the ones from Sacramento and Oakland. Additionally, NIDA’s 95% confidence ellipse mostly lies within the Sacramento and Oakland ellipses. In other words, the full variation in cannabinoids from NIDA can be found in Oakland and Sacramento. However, the full variation from Sacramento and Oakland is not captured by the NIDA varieties. Therefore across all cannabinoids, the government source of *Cannabis* is limited in diversity, not reflecting the range of chemotypes widely available to consumers in state markets. Additionally, we established with the k-means cluster analysis that the best number of clusters given the data from the PCA analysis was two. These two groups are clearly portrayed in the PCA graph with PC1 against PC2 (Figs 4 and S2), revealing that NIDA’s samples are present only in one of the clusters. Therefore, the cannabinoid diversity from the private market is represented in both clusters, while that from the federal cannabinoids is almost entirely found only in one of the clusters, demonstrating again their lack of variation.

Finally, NIDA’s *Cannabis* contains a higher proportion of Cannabinol (CBN) compared to Oakland (P < 0.0001) and Sacramento (P < 0.0001), but Oakland and Sacramento did not differ from each other (P = 0.13).

## Discussion

The objective of this research was to determine whether *Cannabis* produced and sanctioned by the United States federal government for research purposes reflects the *Cannabis* that is widely available in state regulated markets. The data demonstrate that *Cannabis* plants currently grown for NIDA are not representative of plants consumed by recreational and medicinal users through state-legalized markets across the nation. *Cannabis* flower available from dispensaries appears to be more potent and diverse in cannabinoid content.

The illicit Cannabis contains a higher percent of THC compared to NIDA, but lower compared to the legal markets. However, the black market’s CBD is lower than both NIDA and the private market^5^. Furthermore, our results suggest that the private markets also store their Cannabis in conditions that avoid degradation of THC to CBN^32^,^33^,^34^. However, the black market’s CBN percentage appears to be higher than NIDA’s^5^. These CBN measurements could be elevated due to storage time and conditions if the measurements were not done immediately after seizure. Thus, the black market material that is illicitly consumed might have lower CBN than what has been reported^5^.

The cannabinoid levels in NIDA and the state markets differ in several ways. Indeed, THC levels on average in NIDA were 27–35% of those in the state markets, while CBN levels are 11–23 times higher. Studies that have examined the impact of *Cannabis* potency have suggested linear dose dependent effects of THC on outcomes such as impaired driving and cognitive abilities, even at much lower potencies than those reported here^35^,^36^. Other data suggest that the effects of particular cannabinoids (e.g. THC) differ depending on which other cannabinoids (e.g. CBD) are present in the variety consumed^12^. Thus, the effects of *Cannabis* differ by the potency and variety of *Cannabis* tested. Our findings of different cannabinoid profiles in NIDA vs legal market varieties underscore the importance of assessing the impact of *Cannabis* as it is used in the real world on outcomes of high importance to public health^4^.

This underrepresentation of cannabinoid variation and potency in the NIDA’s cultivars should be considered for US investigation in several areas, including chemistry, biochemistry, genomics, biology, psychology, but particularly for medical research. These data suggest that the NIDA varieties underrepresent the variation of cultivars with higher cannabinoid levels and the variation that is found in state-legalized markets^37^,^38^.

Medical research using only a limited number of varieties can be misleading, because variation in the amounts and ratios of cannabinoids may have a significant impact on the outcomes of the studies. Particularly as cannabinoids can have dramatic opposing effects and complex interactions with each other^11^,^12^,^14^,^15^,^16^,^17^,^18^,^19^,^20^,^21^, investigations that only use one source of material may hinder our understanding of pharmacological and therapeutic effects of *Cannabis.* Given that the effects of *Cannabis* may vary depending upon the dose^39^,^40^, where both the chemotype and the dose are important in the medical effects^41^,^42^, and the potential harmful effects^12^, it is crucial for scientific and medical investigations to access the varieties used by patients and recreational users.

Studies reporting on effects of *Cannabis* using NIDA varieties will continue to suffer in terms of external validity, possibly underestimating the effects of more potent varieties that are widely available. Compounding this issue is the fact that the public availability of high-potency *Cannabis* has increased in recent years^1^,^5^. Given our data and recent reviews that have suggested that the greater potency of today’s *Cannabis*, compared to earlier decades^4^, may lead to significantly greater levels of intoxication and possibility of harm, it is important for research to begin understanding consequences and impact of using the publicly available *Cannabis*.

The knowledge gap between what we know from studies using government grown *Cannabis* and ***what we should know*** about the effects of *Cannabis* in the real world could continue to widen with the progressive decriminalization and accessibility of high-potency, dispensary-grade *Cannabis*. This problem can only be addressed by establishing legal methods for US scientists to access *Cannabis* more similar to what is sold and consumed in state-regulated markets.

Despite this being one of the most complete cannabinoid analyses to date, it has a number of limitations. First, data analyzed in this study were collected by separate facilities, NIDA and Steep Hill, which may introduce biases. Inter-laboratory comparative analysis between different methods of testing *Cannabis* products (e.g., different equipment used for this comparison and the various other facilities that offer chemotype testing) has been limited. This limitation is largely imposed by federal laws that prevent third parties (e.g., universities) from conducting such studies. Lastly, Steep Hill data only includes varieties tested at their locations and are not necessarily representative of all *Cannabis* available to consumers. However, with 2980 samples tested, common varieties are well represented. Similarly, our analysis includes the potential current pool of varieties listed as available by the government for research purposes; however the *Cannabis* varieties produced historically by NIDA (and used in NIDA-funded studies published prior to 2012, when these additional NIDA varieties became available) are far less potent. Thus, while our analysis focuses on currently available varieties, the discrepancy between publicly-available *Cannabis* and that used in most existing research is even greater than what we report here.

Moreover, this analysis is limited to the six cannabinoids reported for the NIDA varieties, while additional compounds are known to be important^2^,^8^,^9^. Chemical analyses of *Cannabis* in commercial testing labs include numerous cannabinoids and terpenoids, which vary between lineages^43^ and have important physiological effects^2^,^8^,^9^. Compounds not reported for the NIDA varieties may represent additional important differences between the federally approved cultivars and more widely-used material. However, it is worth noting that the cultivars available through NIDA may compare favorably to individual dispensaries in terms of diversity of cannabinoid levels and ratios of THC to CBD.

Additionally, *Cannabis* flower is one form of *Cannabis* available in state regulated markets, with concentrates and edibles also widely used. It is critical to note that as of May 2016 on the federal website, there is no source of concentrates or edibles for research. Therefore, there is almost no research on the effects of cannabinoids in extract or edible form, even though in Colorado alone, approximately 650,000 edible units are sold each month. It may be a challenge for the federal government to produce *Cannabis* in a way that reflects the diversity of products used by the public in states where it is legal.

In conclusion, this study offers a comparison between six cannabinoids from *Cannabis* produced in four cities in the US and the NIDA supply farm. The data demonstrate that *Cannabis* produced by NIDA is both less diverse in variety and less potent in the amount of cannabinoids. Because most federally approved research requires the use of government produced *Cannabis*, this mismatch between what the public is using and what is available to researchers limits scientific study on the potential harms or benefits. In recent years, federal sources have pursued diversification of their varieties with a goal of increasing the diversity and potency of research *Cannabis*. The research presented here provides concrete data that can inform further changes, so that *Cannabis* available to researchers in the future can better reflect the types of products widely-used by the public.

## Methods

Cannabinoids from multiple varieties of the species *Cannabis sativa L.* in four cities of the US Denver, Oakland, Sacramento, and Seattle were measured by Steep Hill Labs, Inc. during October to December of 2013 and January to September of 2014. These samples were not randomly chosen for two main reasons: First, because we rely on the locations where Steep Hill has facilities, and second, because even though dispensary owners and *Cannabis* producers are required by law to test their product in some of those jurisdictions, it is a choice to select between the multiple companies that provide these services. However, Steep Hill is the only company that tests for 17 cannabinoids and ten terpenes^43^,^44^, and has multiple facilities in cities where *Cannabis* is legal, medically and/or recreationally. The use of the same testing procedures across multiple marketplaces allows us to compare cannabinoid levels among the largest state markets. Moreover, this dataset includes many widely used varieties, as well as minor ones.

Cannabinoid measurements are performed on the flower of female plants, where most of the cannabinoids are produced^45^,^46^. Approximately 500 milligrams of each sample was extracted into methanol, filtered, diluted 1:20, and analyzed by HPLC, using a mobile phase consisting of 0.1% formic acid in water and 0.1% formic acid in methanol. The gradient started at 72% methanol and ended at 99% methanol, with a total run time including equilibration of approximately 17 minutes. Cannabinoid standards purchased from Cerilliant (Round Rock, TX), RESTEK (Bellefonte, PA), Lipomed (Cambridge, MA), and Restek (Bellefonte, PA), were used to create linear, eleven point calibration curves (0.5–1000 ppm) to allow quantification of cannabinoids over three to four orders of magnitude. C18 columns purchased from RESTEK (Raptor ARC-18, Bellefonte, PA) or Phenomenex (Kinetex C18, Torrance, CA) were used for chromatographic separation. For samples measured in 2012/2013, concentrations of cannabinoids without commercially available standards were estimated using published absorptivies^47^. Commercial standards have been available for each of the cannabinoids included in the study since at least 2014. Column analyte measurements were reported as percent mass in each sample and is not corrected for moisture content. All Steep Hill samples were measured using liquid chromatography (LC), in Denver with Agilent LC equipment, in Seattle and Sacramento using Shimadzu LC equipment, and in Oakland using both types of machinery.

The data from NIDA were obtained from their website on November 15, 2015^31^. Details about data collection or the equipment used was not currently specified. Total sample sizes for each of the cannabinoids by location are given in Table 1. Even though Steep Hill measures additional cannabinoids, our analyses focused on cannabinoids shared between the NIDA and SteepHill datasets (N = 6). NIDA uses gas chromatography for their analysis^48^,^49^,^50^, which only measures the neutral form of the cannabinoids. Thus, to allow comparison of the data in the same form, the acidic form of each cannabinoid measured by HPLC was transformed into the neutral form by multiplying each LC value by the ratio of molecular masses of the neutral cannabinoid to the acidic cannabinoid, which represents the mass ratio of the neutral relative to acidic forms after decarboxylation. The mandated value for this conversion according to several US states is approximately 0.88 and states such as Washington^51^ and Nevada^51^ mandate reporting of total THC content from HPLC analysis using this calculation, and adding the calculated values to the values measured for the neutral form. The final result is equivalent to the measurements taken by NIDA. We also performed a separate analysis using the conversion factor reported by Dussy and collaborators of 0.68^51^. Even though both datasets using the two conversion rates differ from each other, the overall results and conclusions are the same: a significantly lower total diversity and total level of cannabinoids from the NIDA samples. Therefore, we are only presenting the sample sizes and results using the state-mandated conversion rate of 0.88.

In order to analyze the cannabinoid composition information across the sites, we first selected only those tests that demonstrated 1% or greater concentration for the specific cannabinoid under analysis. This method allowed us to more accurately report the concentration of each cannabinoid across varieties, many of which are bred for high levels of a single cannabinoid at the expense of other cannabinoids (e.g., high THC, low CBD or visa versa). Due to the absence of samples that produced more than 1% CBN and CBC, we excluded these two cannabinoids from the analysis. An ANOVA was then performed for each cannabinoid with location as a factor and a posterior post-hoc analysis using Tukey, except for THC-V where we used Bonferroni (Fig. 1). We determined the cannabinoid range on a box and whiskers plot (Fig. 2) to visualize the array, median, minimum, and maximum of each compound by location. Additionally, samples producing greater than 1% by mass for both THC and CBD were identified (Fig. 3), indicating functional copies of both THCA and CBDA-synthases, and THC:CBD ratios were calculated. The THC:CBD ratios were used to perform four F-tests comparing NIDA samples to samples from the other four locations. To further understand the variation between locations in their overall cannabinoid composition, we performed Principal Components Analysis (PCA; Fig. 4) with the Car package^52^ from R statistical software. For this analysis, all samples, including the ones that produced less than 1% cannabinoids, were used; however, both Denver and Seattle were excluded from the PCA due to the absence of CBN and CBC. The total sample size for this PCA is given in Table 1. We used the same dataset from the PCA to calculate k-means clustering to understand the number of partitions and their means given our data. Finally, with the three locations that contained CBN data, Sacramento, Oakland and NIDA, we performed an ANOVA and a posthoc analysis to compare the proportion of CBN from the total cannabinoids. We used the R statistical framework to perform all analyses. All code is available on https://github.com/KaneLab/Bioinformatics-Scripts/blob/master/scientific_reports_code.Rmd.

## Additional Information

**How to cite this article**: Vergara, D. *et al*. Compromised External Validity: Federally Produced *Cannabis* Does Not Reflect Legal Markets. *Sci. Rep.* **7**, 46528; doi: 10.1038/srep46528 (2017).

**Publisher's note:** Springer Nature remains neutral with regard to jurisdictional claims in published maps and institutional affiliations.

## Supplementary Material

Supplementary Information

## Figures and Tables

**Table 1:** Sample sizes.

	Denver		NIDA			Oakland			Sacramento			Seattle	
	TOTAL	>1	TOTAL	>1	PCA	TOTAL	>1	PCA	TOTAL	>1	PCA	TOTAL	>1
CBD	1141	42	98	56	90	755	110	481	981	101	981	103	4
CBN	—	—	98	—	90	481	—	481	981	—	981	—	—
THC	1141	1112	98	64	90	755	692	481	981	952	981	103	103
CBG	992	98	96	1	90	481	41	481	981	259	981	103	12
THC-V	992	12	96	—	90	481	6	481	981	21	981	103	1
CBC	—	—	96	—	90	481	—	481	981	2	981	—	—
THC & CBD >1	21	—	24	—	—	77	—	—	81	—	—	4	—

Sample sizes for each cannabinoid at the different locations. Denver and Seattle lack samples for CBN and CBC. The first column represents the total sample sizes for each cannabinoid, the >1 column represents the number of samples that produced more than 1% content of each cannabinoid. No location had varieties that produced >1% CBN or CBC. The last column represents the sample sizes used for the PCA, which was performed only with samples from Oakland, Sacramento, and NIDA. The last row represents the samples that produce more than 1% for both THC and CBD.
