# Supplementary material for: Compromised External Validity: Federally Produced Cannabis Does Not Reflect Legal Markets
Source: Sci Rep. 2017 Apr 19;7:46528. doi: 10.1038/srep46528 (PMC5395929; doi:10.1038/srep46528)
Supplement: Supplementary Information [file srep46528-s1.pdf]

Supplementary Information

Title: Compromised External Validity: Federally Produced *Cannabis* Does Not Reflect Legal Markets

Authors: Daniela Vergara[1]*, L. Cinnamon Bidwell[2], Reggie Gaudino[3], Anthony Torres[3], Gary Du[3], Travis C. Ruthenburg[3]†, Kymron deCesare[3], Donald P. Land[3], Kent E. Hutchison[4] and Nolan C. Kane[1]*

Results

The F-tests between NIDA and the other four localities are statistically significant: NIDA vs. Denver, $F=0.11$, $P<0.001$; NIDA vs. Oakland, $F=0.03$, $P<0.001$; NIDA vs. Sacramento, $F=0.004$, $P<0.001$; NIDA vs. Seattle $F=0.191$, $P<0.05$. The median of the THC/CBD ratio is similar between localities, but the range (25th, 75th percentile, maximum, and minimum) are greater for most localities particularly Oakland and Sacramento (fig. S1). Additionally, Sacramento, Oakland, and Denver have samples with a much higher ratio, demonstrating that all of these localities have higher variety compared to NIDA. Indeed, among the samples with both THC and CBD, NIDA's variance for the THC/CBD ratio is the lowest (0.021), followed by Seattle (0.11), Denver (0.18), Oakland (0.755) and Sacramento (6.07).

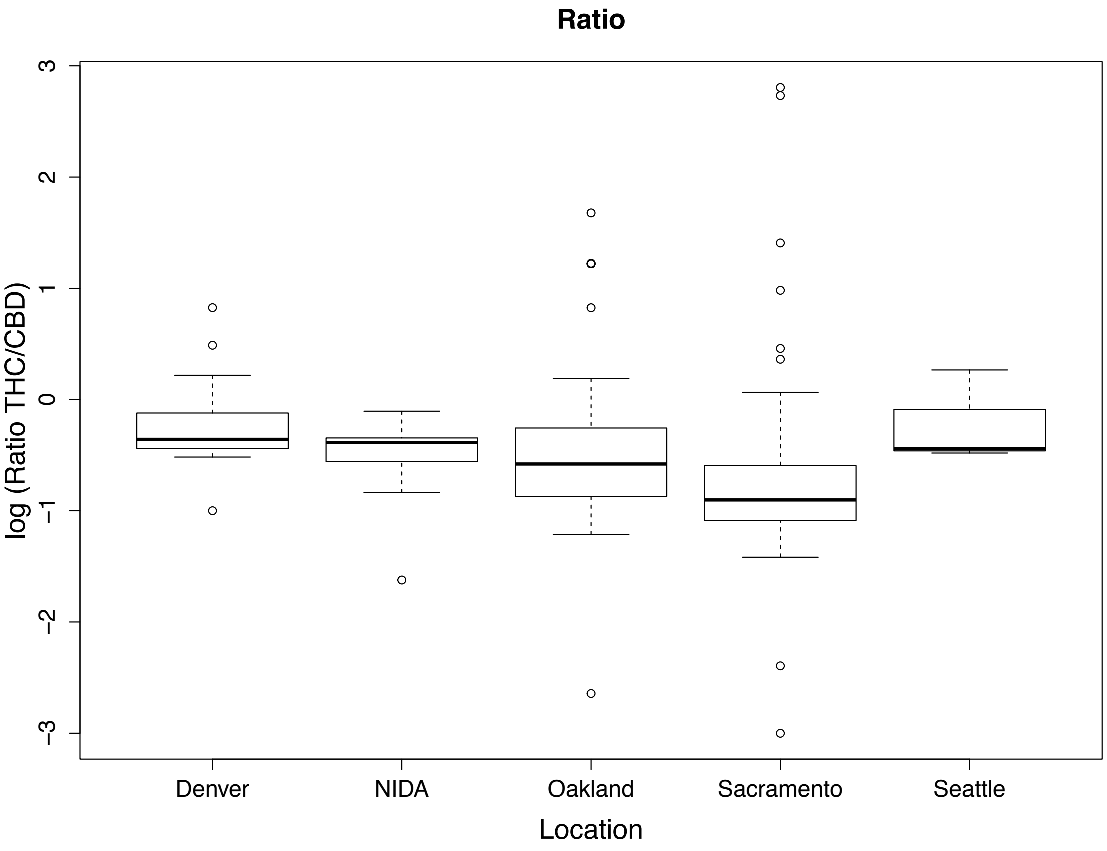

Fig. S1. Median and range for the log transformed THC to CBD ratio by location. Median (line within the box), 25th and 75th percentile (bottom and top of the box respectively), and range (bars outside the box). Outliers are dots outside the box and range.

With the k-mean clustering we established the samples we analyzed can be clustered into two groups based on similarities in the six measured cannabinoids. These two groupings (fig. S2) show that NIDA's cannabinoid variation is found almost entirely in one of the two clusters, while the private market's variation is found throughout both clusters. This finding further supports that the federal government does not capture the diversity in cannabinoids from the private market.

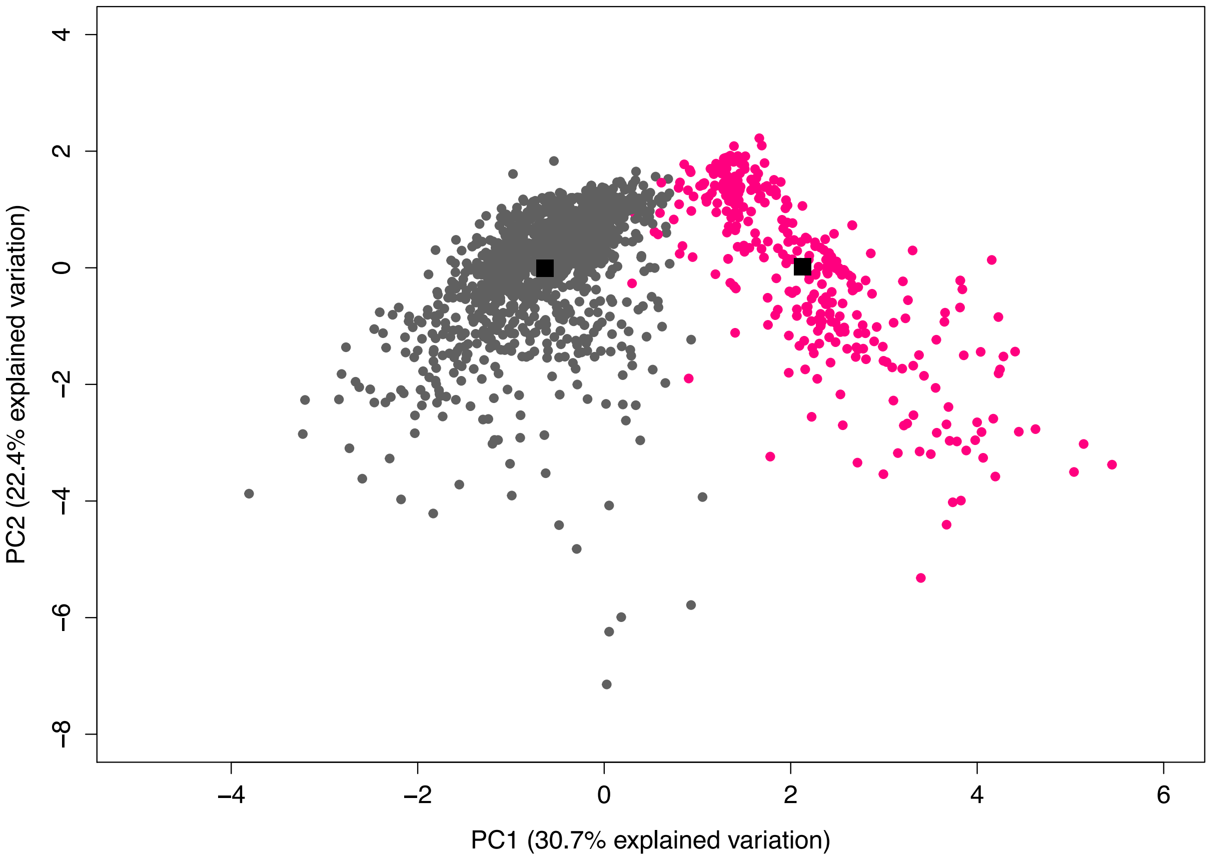

Fig. S2. Clusters from PC1 vs PC2 for three locations. Two clusters represent the variation given our data in gray and magenta. The black boxes represent the means of each cluster.
